# Therapeutic Plasma Exchange Decreases Levels of Routinely Used Cardiac and Inflammatory Biomarkers

**DOI:** 10.1371/journal.pone.0038573

**Published:** 2012-06-07

**Authors:** Oktay Tutarel, Paulina Golla, Gernot Beutel, Johann Bauersachs, Sascha David, Bernhard M. W. Schmidt, Ralf Lichtinghagen, Jan T. Kielstein

**Affiliations:** 1 Department of Cardiology and Angiology, Hannover Medical School, Hannover, Germany; 2 Department of Nephrology and Hypertension, Hannover Medical School, Hannover, Germany; 3 Department of Hematology, Hemostaseology, Oncology and Stem Cell Transplantation, Hannover Medical School, Hannover, Germany; 4 Department of Clinical Chemistry, Hannover Medical School, Hannover, Germany; University of Milan, Italy

## Abstract

**Background:**

Therapeutic plasma exchange (TPE) plays a key role in the management of various diseases, from thrombotic thrombocytopenic purpura and Goodpasture's syndrome to cardiac allograft rejection. In many of these disease states cardiac and inflammatory involvement is common and biomarkers are routinely used for diagnosis or assessment of therapeutic success. The effect of TPE on biomarkers used in the clinical routine has not been investigated.

**Methods:**

TPE was initiated for established clinical conditions in 21 patients. Troponin T, NT-proBNP, C-reactive protein, procalcitonin and routine chemistry were drawn before and after TPE, as well as before and after the 2^nd^ TPE. The total amount of these markers in the waste bag was also analyzed.

**Results:**

In 21 patients 42 TPEs were performed. The procedure reduced plasma levels of the examined biomarkers: 23% for NT-proBNP (pre vs. post: 4637±10234 ng/l to 3565±8295 ng/l, p<0.001), 64% for CRP (21.9±47.0 mg/l vs. 7.8±15.8 mg/l, p<0.001) and 31% for procalcitonin (0.39±1.1 µg/l vs. 0.27±0.72 µg/l, p=0.004). TPE also tended to reduce plasma levels of troponin T by about 14% (60.7±175.5 ng/l vs. 52.2±141.3 ng/l), however this difference was not statistical significant (p=0.95). There was a significant correlation between the difference of pre TPE levels to post TPE levels of all examined biomarkers and the total amount of the removed biomarker in the collected removed plasma.

**Conclusions:**

TPE significantly reduces plasma levels of inflammatory and cardiac biomarkers. Therefore, post TPE levels of cardiac and inflammatory biomarkers should be viewed with caution.

## Introduction

Therapeutic plasma exchange (TPE) is an extracorporeal blood purification technique that was first described in 1914 [1]. It removes pathogenic substances such as autoantibodies, lipoproteins and circulating immune complexes from the plasma [2]–[4]. According to the 2010 guidelines of the American Society of Apheresis TPE plays a key role in the management of various diseases and is the treatment of choice for acute ANCA associated rapid progressive glomerulonephritis, thrombotic thrombocytopenic purpura, Guillan-Barré syndrome, Goodpasture's syndrome and cardiac allograft rejection to name a few [3]–[4]. Most recently TPE was the mainstay of therapy during the enterohemorrhagic E. coli associated outbreak of hemolytic uremic syndrome in Germany [5]. In many of the aforementioned disease states cardiac and inflammatory involvement is common. Patschan et al. [6] showed that about one out of five patients with thrombotic microangiopathy suffers from acute myocardial infarction. Moreover, Dengler et al. [7] found that acute allograft rejection after human heart transplantation is often associated with increased serum concentrations of troponin. Despite the pivotal role of biomarkers in the diagnosis and therapy of heart disease [8], [9], the effect of TPE on circulating biomarkers has not been investigated. Therefore, the aim of our study was to assess the effect of TPE on the plasma concentration of cardiac biomarkers and inflammatory markers in patients undergoing TPE using albumin as replacement fluid. In addition to the blood levels, the total amount of the removed biomarker was determined in the collected eliminated plasma.

**Table 1:** Clinical characteristics of study population.

Age (yrs)	51.6±13.5
Female	10 (47.6%)
Male	11 (52.4%)
Body mass index (kg/m^2^)	25.1±5.0
*Underlying illness necessitating plasmapheresis*	
humoral rejection after solid organ transplantation	8
Guillan-Barré syndrome and variants	3
monoclonal gammopathy	2
multiple sclerosis	2
rapid progressive glomerulonephritis	1
Polyneuritis	1
microscopic polyangitis	2
Cryoglobulinemia	1
cytotoxic antibodies	1

Data are expressed as mean±SD or as counts.

## Materials and Methods

The study was approved by the local ethics committee at the Hannover Medical School. All patients gave written informed consent.

**Figure 1:**
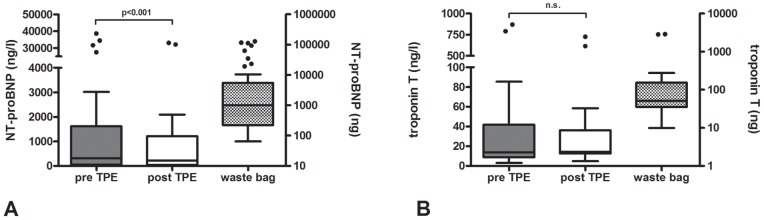
Plasma levels of cardiac biomarkers. Pre and post TPE (left axis) and total amount in the waste bag (right axis). A. N-terminal-pro-brain natriuretic peptide (NT-proBNP); B. troponin T.

TPE was initiated for various diseases in 21 patients **(** **Table 1** **)**. Blood samples for measurement of plasma troponin T, NT-proBNP, C-reactive protein, procalcitonin and routine chemistry were drawn before and at the end of the TPE, before the rinse back of the blood. Also the total amount of the measured biomarkers in the waste bag was analyzed. All measurements were done with routine laboratory tests using certified assay methods.

Statistical analysis was performed with SPSS 15.0 and GraphPad Prism 5. Continuous data were presented as means ± standard deviation and median + interquartile range (IQR). Categorical data are presented as counts and proportions. Comparisons before and after the 1^st^ and 2^nd^ TPE as well as post 1^st^ TPE levels and pre 2^nd^ TPE levels were examined using the Wilcoxon test. For correlation Spearman's correlation coefficient was calculated. The significance level was set at p<0.05.

**Figure 2:**
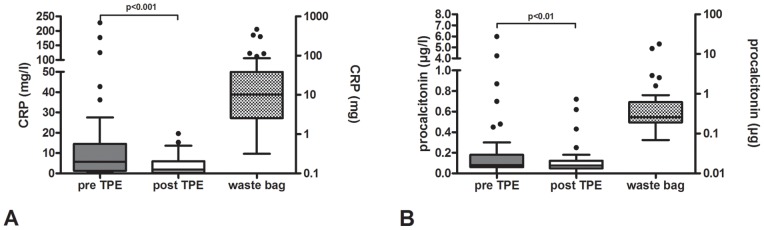
Plasma levels of inflammatory biomarkers. Pre and post TPE (left axis) and total amount in the waste bag (right axis). A. C-reactive protein (CRP); B. procalcitonin.

**Figure 3:**
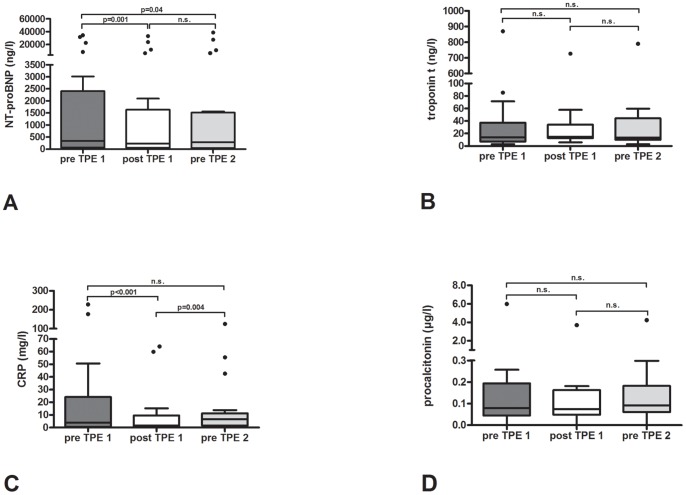
Longitudinal kinetics of the investigated biomarkers. A. N-terminal-pro-brain natriuretic peptide (NT-proBNP); B. troponin T; C. C-reactive protein (CRP); D. procalcitonin.

## Results

In a total of 21 patients 42 TPEs were performed. **Table** **1** shows the clinical characteristics of the study population.

The reduction of the examined biomarkers comparing pre TPE and post TPE plasma levels was 23% for N-terminal-pro-brain natriuretic peptide (NT-proBNP) (pre TPE vs. post TPE: 4637±10234 ng/l (median 310 ng/l, IQR 1569 ng/l) to 3565±8295 ng/l (median 219 ng/l, IQR 1168 ng/l), p<0.001), 64% for C-reactive protein (CRP) (21.9±47.0 mg/l (median 5.7 mg/l, IQR 13.25 mg/l) vs. 7.8±15.8 mg/l (median 1.9 mg/l, 5.6 IQR mg/l), p<0.001) and 31% for procalcitonin (0.39±1.1 µg/l (median 0.08 µg/l, IQR 0.12 µg/l) vs. 0.27±0.72 µg/l (median 0.08 µg/l, IQR 0.07 µg/l), p=0.004). TPE also tended to reduce plasma levels of troponin T by about 14% (60.7±175.5 ng/l (median 13.7 ng/l, IQR 33.0 ng/l) vs. 52.2±141.3 ng/l (median 14.2 ng/l, IQR 23.6 ng/l), however this difference was not statistical significant (p=0.95) (**Figure** **1** **and** **2**).

There was a significant correlation between the difference of pre TPE to post TPE levels and the total amount of the removed biomarker in the collected eliminated plasma for NT-proBNP (r=0.821, p<0.001), troponin T (r=0.694, p<0.001), CRP (r=0.994, p<0.001) and procalcitonin (r=0.504, p<0.001).

The absolute amount of the removed biomarkers was 15215±34269 ng (median 1014 ng, IQR 5289 ng) for NT-proBNP, 216.6±599.7 ng (median 51.5 ng, IQR 118.7 ng) for troponin T, 46.6±97.8 mg (median 10.3 mg, IQR 35.8 mg) for CRP, and 1.23±3.44 µg (median 0.26 µg, IQR 0.43 µg) for procalcitonin (**Figure** **1** **and** **2**). Pre and post TPE hematocrit did not differ (34.2±5.1% vs. 34.5±6.0%; p=0.442), excluding hemoconcentration or -dilution, which could have influenced the concentration of biomarkers.

The kinetics of the biomarkers were analyzed via a comparison between pre TPE (pre TPE1) and post TPE (post TPE 1) levels for the first TPE with the pre TPE levels before the second TPE (pre TPE 2). The second TPE was performed 25±5 hours after the first. While NT-proBNP increased 1 day after the first TPE (pre TPE 2 vs. post TPE 1 (4270±10097 ng/l (median 284 ng/l, IQR 1470 ng/l) to 3839±8746 ng/l (median 227 ng/l, IQR 1586 ng/l)), it was still significantly reduced compared to pre TPE 1 levels (5003±10606 ng/l (median 336 ng/l, IQR 2336 ng/l), p=0.04) (**Figure** **3**). A different effect was seen for CRP. Pre TPE 2 levels were higher than post TPE 1 levels (15.3±28.8 mg/l (median 6.6 mg/l, IQR 9.8 mg/l) vs. 9.3±18.2 mg/l (median 1.5 mg/l, IQR 9.45 mg/l), p=0.004) and statistically not significant lower than pre TPE 1 levels (28.5±60.0 mg/l (median 3.9 mg/l, IQR 23.6 mg/l), p=0.211) (**Figure** **3**). For procalcitonin and troponin T no significant difference was observed in the longitudinal analysis (pre TPE 1 vs. post TPE 1 vs. pre TPE2 for procalcitonin 0.42±1.29 µg/l (median 0.08 µg/l, 0.15 IQR µg/l) vs. 0.30±0.79 (median 0.07 µg/l, 0.11 IQR µg/l) vs. 0.35±0.91 µg/l (median 0.09 µg/l, IQR 0.12 µg/l), and for troponin T 62.3±186.4 ng/l (median 13.8 ng/l, IQR 30.0 ng/l) vs. 55.1±154.6 (median 14.8 ng/l, IQR 21.3 ng/l) vs. 59.1±168.6 ng/l (median 13.6 ng/l, IQR 34.4 ng/l) (**Figure** **3**).

## Discussion

To our knowledge this is the first study evaluating the effect of a single TPE on cardiac and inflammatory biomarkers and directly quantifying the amount of these removed substances. We show that a single TPE can reduce plasma levels of several cardiac and inflammatory biomarkers (i.e. NT-proBNP, CRP and procalcitonin). A similar effect was seen for troponin T, even if it did not reach statistical significance.

In a recent study, Hershcovici et al. reported their experience with TPE in 6 patients with severe hyperlipidemia [10]. CRP levels were significantly reduced. Unfortunately, the authors did not measure CRP levels in the waste bag. Bektas and colleagues reported a significant reduction of serum aminotransferases and bilirubin levels after TPE in patients with liver failure [11]. Even if data for hematocrit were not reported and amount of the circulating markers in the waste bag was not analyzed, the authors suggested hemodilution as a possible mechanism of biomarker reduction in the plasma. From our own data we can rule out hemodilution since the hematocrit remained stable. It is reasonable to assume that TPE removes these biomarkers from the plasma since there is a significant correlation between the difference of pre TPE to post TPE levels and the total amount of the removed biomarker in the collected eliminated plasma. This demonstrates that the observed reduction is indeed a direct consequence of TPE-associated removal, rather than (just) an improvement in the particular disease condition.

Our data are of importance for the interpretation of treatment success in particular in those diseases in which TPE is frequently performed. The decrease of CRP or procalcitonin in Goodpasture syndrome for instance is thought to be associated with a more favorable outcome [12]. However, our results suggest that TPE decreases CRP and procalcitonin. Therefore, we should be cautious when using this marker for the assessment of treatment effects in cases of Goodpasture syndrome (and probably many others) in which TPE is part of the treatment.

In up to 20 percent of patients with thrombotic microangiopathies acute coronary syndromes are observed [6]. TPE lowers troponin T levels and could hinder prompt diagnosis in patients with clinical signs of acute coronary syndrome leading to false negative diagnosis.

Changes in NT-proBNP levels have been proposed as an approach to detect rejection in cardiac transplant recipients [13]. Again, TPE is part of the treatment in patients with humoral rejection [14]. According to our results NT-proBNP levels could be false low after TPE possibly masking ongoing rejection.

Longitudinal analysis reflecting the kinetics of the biomarkers after TPE showed that while the reduction in CRP is not significant after one day, the reduction of NT-proBNP lasts.

A limitation of our study is that albumin was used as replacement fluid during TPE because fresh frozen plasma would have interfered with the analysis of the biomarkers. Therefore, our results are valid for TPE with albumin. Further, underlying illnesses necessitating TPE spanned a variety of conditions. Different morbidities may affect the kinetics of the measured biomarkers. This could lead to an over- or underestimation of the effects of TPE. In future studies a more homogenous patient group should be studied and ideally be compared with an untreated control group.

In conclusion, post TPE levels of cardiac and inflammatory biomarkers especially NT-proBNP should be viewed with caution.
